# Prevention and control of avian influenza virus: Recent advances in diagnostic technologies and surveillance strategies

**DOI:** 10.1038/s41467-025-58882-4

**Published:** 2025-04-15

**Authors:** Qian Niu, Zhiwen Jiang, Lifang Wang, Xiang Ji, Guy Baele, Ying Qin, Liyan Lin, Alexander Lai, Ye Chen, Michael Veit, Shuo Su

**Affiliations:** 1 Department of Laboratory Medicine/Clinical Laboratory Medicine Research Center, West China Hospital, Sichuan University, Chengdu, China; 2 Engineering Laboratory of Animal Immunity of Jiangsu Province, College of Veterinary Medicine, Academy for Advanced Interdisciplinary Studies, Nanjing Agricultural University, Nanjing, China; 3 National Key Laboratory of Veterinary Public Health Security, College of Veterinary Medicine, China Agricultural University, Beijing, China; 4 Department of Mathematics, School of Science and Engineering, Tulane University, New Orleans, LA USA; 5 Department of Microbiology, Immunology and Transplantation, Rega Institute, Laboratory for Clinical and Epidemiological Virology, KU Leuven, Leuven, Belgium; 6 Department of Biological and Physical Sciences, College of Agriculture, Health, and Natural Resources, Kentucky State University, Frankfort, KY USA; 7 Key Laboratory of Fujian-Taiwan Animal Pathogen Biology, College of Animal Sciences, Fujian Agriculture and Forestry University, Fuzhou, China; 8 Institute for Virology, Veterinary Faculty, Free University Berlin, Berlin, Germany

**Keywords:** Virology, Molecular medicine, Policy and public health in microbiology, Influenza virus

## Abstract

The natural host for avian influenza virus (AIV) is waterfowl. However, certain subtypes have breached species barriers, causing epizootics in many avian and mammalian species with occasional zoonotic infections in humans. The ongoing spread of highly pathogenic avian influenza (HPAI) A(H5N1) poses a significant and growing public health threat. Here, we discuss recent advances in viral detection and characterization technologies and their integration into the diagnostics and surveillance of AIV within a “One Health” framework.

Avian influenza virus (AIV), a member of the *Orthomyxoviridae* family, is an influenza A virus (IAV) with a unique ecology^1^. Currently, 16 H subtypes (H1–H16) and 9 N subtypes (N1–N9) have been identified based on the antigenicity of hemagglutinin (HA) and neuraminidase (NA)^2^. AIVs are further classified into high pathogenicity (HP) and low pathogenicity (LP) based on the amino acid sequence of the HA protein cleavage site. HPAIVs, typically of the H5 or H7 subtypes, possess a multi-basic cleavage site, enabling systemic infection, while LPAIVs have a monobasic site, limiting replication to specific tissues. Waterfowl are the natural hosts, but AIV frequently crosses species barriers, infecting various mammals, including humans^3–6^. Several subtypes, including H3N8, H5N1, H7N9, H9N2, H10N5, and many more, have infected humans, mainly through direct contact with infected animals or contaminated environments^7^. Though interspecies transmission barriers exist, some subtypes have adapted to new hosts via mutations and reassortment, leading to epizootics, enzootics^8^, and even pandemics.

The recent global outbreaks of the highly pathogenic avian influenza (HPAI) H5N1 virus underscores its evolving transmission dynamics. The zoonotic potential of HPAIV H5N1 was first recognized during the 1997 avian influenza outbreak in Hong Kong, which resulted in 18 human cases and 6 fatalities^9^. Since 2022, H5N1 has caused over 70 million poultry losses across five continents, even reaching Antarctica^10^. In March 2024, dairy cows in Texas and Kansas tested positive, with high viral concentrations found in unpasteurized milk^11^. This led to infections in four dairy workers and fatal transmission to two cats, highlighting a multispecies outbreak in the US^12,13^. This interconnected multispecies outbreak underscores the ability of this strain to mutate rapidly and switch between species^14^. The recent severe cases and fatalities resulting from HPAI H5N1 infections have further increased public health concerns. As of January 20^th^, 2025, the WHO has reported 964 human H5N1 cases across 24 countries since 2003, with a fatality rate of 48%^15^. Emerging hosts like alpacas, goats, civets, and minks further highlight the need for comprehensive multi-species surveillance programs to mitigate the rapid spread of H5N1.

Surveillance of AIV in mammals remains inadequate due to limited systematic sampling strategies and socio-economic barriers. Many AIV-infected mammals show mild or atypical symptoms, complicating detection. Establishing structured surveillance programs, improving diagnostic technologies, and training specialized personnel are essential. We emphasize recent advances in diagnostics and surveillance strategies that, when integrated with streamlined monitoring, can significantly enhance AIV control and prevention.

## Diagnosis of AIV infection

Recognizing signs and symptoms is instrumental in the preliminary evaluation of AIV infection. However, clinical manifestations vary greatly, depending on the characteristics of the virus (such as the virulence and subtype) and of the infected subject (such as age, immune status, and any co-infections). AIV (typically HPAIV H5 viruses) infections in humans frequently present non-specific “flu-like symptoms” – typical of respiratory infections. However, uncommon signs and symptoms due to viral replication at unusual infection sites, present a challenge for accurate diagnosis. For example, the dairy workers infected with H5N1 from dairy cows developed conjunctivitis only. Physicians should, therefore, consider patients’ history for any potential exposure include possible AIV infection, during initial differential diagnosis to recognize these uncommon clinical presentations. Likewise, veterinarians should be vigilant in the diagnosis of sick animals. Of note, the diagnosis of cattle infected with H5N1 was triggered by two cats having died after consumption of raw milk from infected dairy cows with atypical signs and symptoms^16^.

**Fig. 1:**
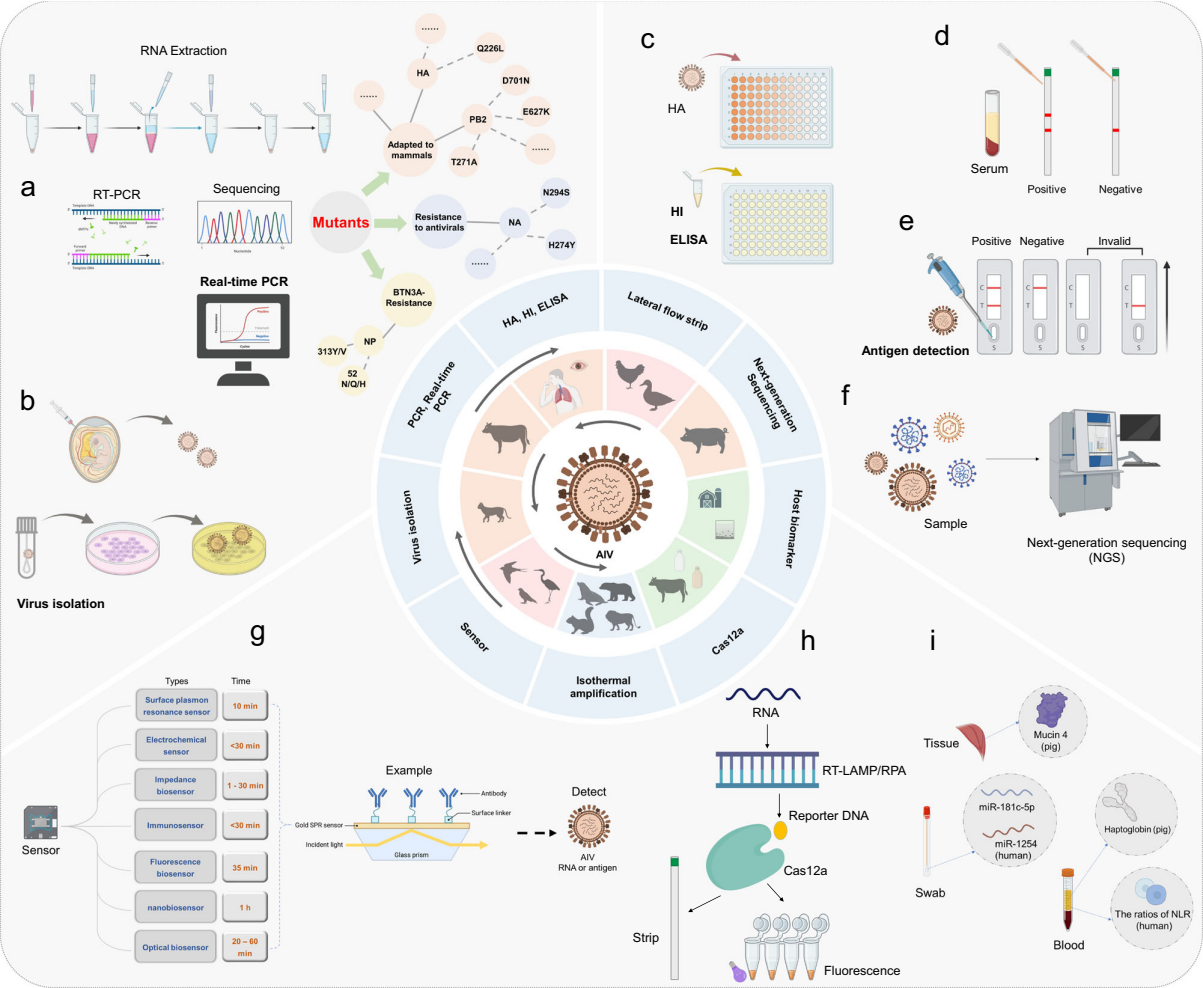
Diagnostic techniques for AIV surveillance and diagnosis of AIV infection. Center panel: A schematic diagram to illustrate the interconnection among the hosts, environment, and food impacted by AIV. Black arrows denoting the direction of viral transmission, such as cattle to cat, cattle to human, and bird to other mammals. The outer circle illustrates the diagnostic techniques that are employed for detecting AIV, including the RNA extraction process. **a** RT-PCR and qPCR, sequencing for identification of AIV mutations, including those adaptation to mammals, drug-resistance, and BTN3A-resistance. **b** AIV isolation through chicken embryos and cell culture. **c** HA or HI assays. **d** Lateral flow test strips for antibody detection. **e** Lateral flow test strips for antigen detection. **f** next-generation sequencing analysis. **g** Different sensing technologies for detection of AIV. **h** Isothermal-amplification detection technologies, including RT-LAMP, RT-RPA, and Cas12a. **i** Identification of host biomarkers post-infection. *All images within this figure are created in BioRender. Jiang, Z. (2025)*.

Laboratories play a critical role in formulating clinical management strategies and guiding epidemic control measures. A summary of laboratory diagnostic technologies for AIV is shown in Fig. 1. Organizations like the WHO^17^, the World Organization for Animal Health (WOAH^18^), and the Centers for Disease Control (CDC^19^) regularly issue updated guidelines for AIV testing and diagnosis. Recommended diagnostic techniques for AIV include virus isolation, antigen detection, real-time or quantitative polymerase chain reaction (qPCR), and serological assays, such as hemagglutination inhibition (HI) and enzyme-linked immunosorbent assay (ELISA). The advantages and disadvantages of these tests, plus newer technologies, are discussed below.

Virus isolation is considered the “gold standard” for diagnosing AIV^19^. However, despite its diagnostic value, it is labor-intensive, dependent on professional and technical expertise, and poses biosafety challenges. It has largely been replaced by direct detection of viral nucleic acids as an initial diagnostic method. These nucleic acids tests (NATs), such as reverse transcription PCR (RT-PCR) and qPCR, are now commonly used for the diagnosis of suspected cases and for routine screening purposes. RT-PCR and qPCR are now recommended by the WHO and the WOAH as an alternative “gold standard”, and these tests had been deployed in national and regional reference laboratories. The advantages of NATs are that they are rapid, easily standardized, requiring minimal instruments and equipment, and, importantly, they can be scaled up by high-throughput platforms and by robotics, which is particularly useful for mass screening during an outbreak. Conserved genes, such as matrix (M) or nucleocapsid protein (NP), are used as targets for qPCR, as initial identification for AIV, followed by a multiplexed qPCR for the HA gene to determine the AIV subtype and whether it is an HPAIV or LPAIV^20,21^. Of note, an optimized dual-target H5 subtype qPCR method has recently been developed using WHO’s H5 AIV primer-probe sequences^22^. This method was designed to cover the diverse viruses within clade 2.3.4.4b while maintaining a high sensitivity for H5 AIV across various clades. Regular assessment of the variations in diagnostic targets is essential for maintaining the accuracy and reliability of detection outcomes.

Antigen-detection tests, such as lateral flow antigen-capture tests by using colloidal gold conjugates, are easy to perform and provide results rapidly. However, they have low diagnostic sensitivity, resulting in false-negative results, which could delay timely mitigations. Diagnostic sensitivity may be improved by using label-free quantum dot (QD) probes, which lowers the detection limit to 0.09 ng/mL^23^. A diagnostic test utilizing QDs conjugated with specific antibodies against IAV subtypes H5 and H9 through amide linkage has demonstrated its potential for rapid and efficient differential diagnosis. This simple test provides objective results within 15 min, highlighting the efficiency of QD-linked fluorescence immunoassay (QD-LFIA). Moreover, this method does not require expensive equipment or sophisticated procedures and is easily converted to high-throughput screening, making it particularly suitable for field applications and/or resource-limited situations^24^.

When a sample tests positive, there is a need to determine the AIV subtype and any associated mutations by sequencing, to identify the source and to formulate mitigation strategies. In the past, Sanger sequencing of viral genomic regions amplified by specific primers, such as the cleavage site in the HA, adaptative mutations^25–27^, E627K, D701N, and T721A in PB2, as well as drug-resistance (e.g., oseltamivir-resistance) mutations like H274Y and N294S in the NA was common practice. However, it has been replaced by next-generation sequencing (NGS), which has the advantage of detecting novel variants providing more comprehensive sequence information at a lower cost. With sequence data obtained through NGS, phylogenetic analysis can provide a more complete picture of viral spatiotemporal evolution and dispersal patterns to estimate public health risks, followed by formulating evidence-based mitigation strategies.

Serological assays identify past viral infections. When serum samples are collected from a large population—humans or animals—and tested by simple screening tests such as ELISA, the result provides a crude estimate of the prevalence of AIV infection, hence identifying at-risk populations. Serological testing has limitations, though, such as the “window period” during the early stage of infection when specific antibodies were not detectable, and the inability to differentiate viral variants. Furthermore, cross-reactivity of antibodies can result in false-positive results. Therefore, interpretation and reliability in using the results in decision-making require extreme caution. Nonetheless, AIV’s ever-expanding range of host species is best evaluated using serological surveillance, such as using competitive ELISA. However, this is hindered by limited research and a lack of necessary reagents, for example, positive and negative controls for specific animal species. Of note, a more rapid and less labor-intensive microneutralization test has replaced the traditional neutralization test for the detection of specific AIV infections in humans and in some animal species.

In addition to the detection of viral antigens and serology, other biomarkers have been explored for the diagnosis of past IAV infection. For example, the expression of host microRNAs (miR-181c-5p and miR-1254) has been shown to correlate with human H1N1 infection^28^. In the diagnosis of pediatric IAV infections, the ratio of neutrophils to lymphocytes had been shown to have sensitivity and specificity of 88.4% and 93.1%, respectively^29^. Other biomarkers have been evaluated as surrogate markers for IAV infection in pigs. For example, porcine mucin-4^30^ and porcine haptoglobin^31^ had been shown to have the potential for the diagnosis of H1N1 infection. These biomarkers are assayed from non-invasive and easily accessible samples such as blood or swabs, which is particularly useful for mass screening. In addition to further validation, the diagnostic accuracy, whether these biomarkers are applicable for other AIV subtypes, and to infections in other host species, has not been determined.

The demand for rapid detection techniques is obvious. Traditional laboratory diagnosis methods often require extended processing times, making them less suitable for real-time diagnosis. The locations of laboratories may not be optimal when the outbreak is at a remote or rural location. On-site virus detection allows prompt identification allowing immediate response. Therefore, there is a need for a rapid, and highly specific and yet sensitive “point-of-care-test” (POCT). POCT does not require expensive instruments and laboratories, and they are being increasingly utilized for rapid detection of AIVs in both clinical (human) and veterinary (animal) contexts. Recently, an isothermal loop amplification-based molecular test with remarkable sensitivity has become available. This test is based on a dual reverse-transcription and recombinase polymerase isothermal amplification (RT-RPA) technology, which detects HA and M2 genes at a low concentration of 1 × 10^−7 ^ng/μL (13.72 copies/μL)^32^. More importantly, these reverse-transcription loop-mediated isothermal amplification (RT-LAMP), or RT-RPA tests, can be deployed without the need for cumbersome equipment. A study has demonstrated a sensitivity that with RT-RPA when combined with CRISPR-Cass12a, the detection limit was lowered to 1.9 copies/μL for blue light and to 1.9 × 10^3^ copies/μL for lateral flow strips^33^. However, designing the primers for RPA analysis remains a significant bottleneck, due to a lack of special primer design software. Using multiple primers in LAMP assays increases the risk of non-specific amplification, thereby limiting target specificity. While specialized sample buffers such as PrimeStore-Molecular-Transport-Medium (Primestore) have significantly improved RNA stability in field-collected samples, POCT platforms offer unique advantages, including rapid detection, ease of operation without the need for specialized personnel, cost-effectiveness, and independence from expensive laboratory equipment. These attributes make POCT a promising future direction for AIV surveillance, particularly in outbreak scenarios where immediate decision-making is required^34^.

Real-time diagnostic techniques based on biosensors offer the advantages of rapidity, high sensitivity, and specificity, and can be engineered as portable instruments. Currently, physical sensors based on optical, electrochemical, and biological (biomarkers, peptides, and bacteriophages) signals are being developed. Micro-array technologies^35^, which can detect and subtype the virus in a single chip, are appealing but costly. Recent advancements have established fluorescence quantitative assays utilizing multi-channel magnetic microfluidic chip screening and aptamer sandwich assays for the detection of H5N1, H7N9, and H9N2 viruses^36^. The detection limits have reached 0.38 TCID_50_/mL for H5N1, 0.75 TCID_50_/mL for H7N9, and 1.14 TCID_50_/mL for H9N2. Recently, the development of sensor-detection technology has not only reduced the detection time but also the cost significantly. For example, a paper-based viral molecularly imprinted sensor has been engineered to detect the H5N1 virus at a remarkable low limit of detection (LOD) of 1.6 × 10^−17^ mol^37^. This paper-based sensor is cost-effective, easy to fabricate, store, and transport, and offers the potential for self-service detection, particularly useful as a mass screening tool. Costs have been further reduced by reusable magnetic surface plasmon resonance (SPR) sensors^38^. A biological sensing scheme using genetically engineered filamentous M13 phage as probes detects H9N2 to high analytical sensitivity of 6.3 copies/mL^39^. These sensor technologies will enhance real-time monitoring and early detection of AIV, allowing timely mitigation to reduce the risk of outbreaks. However, despite the promise of these technologies, significant limitations remain. For example, paper-based microarray technology is unsuitable for field detection due to its complex design and susceptibility to interference by environmental factors. Since SPR relies on the measurement of reflected light, turbid samples may adversely affect the detection accuracy. A small hand-held portable sequencer (MinION) utilizing third-generation sequencing technology is ideal for virus detection and real-time sequencing analysis in field settings^40^. However, the complexity of the nucleic acid extraction process deserves further optimization. Also, there are challenges associated with on-site data analysis, e.g., lack of internet access and insufficient computing power. Improvements in these areas are necessary to enhance their effectiveness and reliability for in-situ applications. Furthermore, engagement with the commercial sector is critical for transforming “promising solutions” into “real diagnostic solutions”.

In summary, the utilization of integrated detection methodologies tailored to specific requirements represents a pivotal approach to monitor the progression of the epidemic and to institute mitigation. In addition, the quality and the timing of sampling from the host—incubation, symptomatic, or convalescence—affect the test results. These factors should be considered for a holistic assessment. With the rapid advancement of artificial intelligence (AI) technology, integration of AI in fields such as synthetic biology is poised to provide a platform for more effective development of next-generation diagnostics^41^. Support and validation from organizations such as the WHO, the WOAH, the FAO, and local reference laboratories are necessary to fully utilize this emerging platform.

## Surveillance and streamlined strategies for AIV control

When an incursion of AIV occurs, the major focus is to contain and to “stamp out” the virus to prevent further adaptive evolution, host infections and mortality^42^. To accomplish this objective, we propose adopting a comprehensive monitoring approach, which aims to mitigate zoonotic disease outbreaks by integrating human and animal health, and environmental factors, to safeguard public health (One Health).

**Fig. 2:**
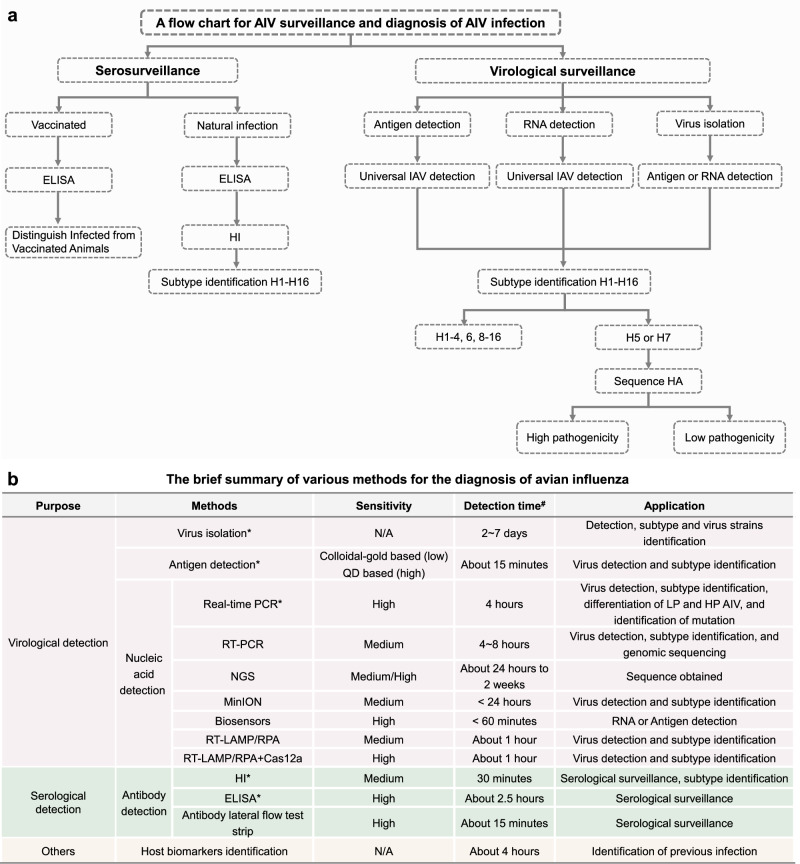
Flow chart for AIV surveillance and diagnosis, and a brief summary of various diagnostic tests. **a** Flow chart: virological surveillance and serological testing. Virological surveillance is employed to detect the presence of AIV through virus isolation and characterization, or the detection of viral proteins or nucleic acids, followed by pathotyping via PCR or sequencing. Serological testing, which detects the antibodies against AIV via methods such as ELISA or HI, holds significance in analyzing past infections or vaccine-induced immunity. **b** Summary of the methodologies in the detection and identification of AIV. *Represents the targeted approach recommended by the WHO and the WOAH. # Represents the detection time, which was determined based on the reference time provided by the WOAH, the CDC, and relevant references.

Given the extensive impact of AIV across multiple domains, the detection and monitoring of AIV during epidemics and spread should be conducted with comprehensive and multifaceted methodologies by including both virological and serological surveillance, as shown in the flow chart (Fig. 2a). The complex human-animal-environment interface can be assessed more accurately. Virological surveillance includes surveillance for AIV from the environment (including lake and aquatic systems, agricultural areas, sewage systems, animal production facilities, and indoor air), from diverse animal populations (including wild birds, companion animals, stray animals, poultry, and livestock), from the human population (both at-risk groups and the general public), and from food products to achieve early detection. Such a comprehensive monitoring approach aligns with the One Health framework, allowing early detection of emergent strains, such as the presence of adaptive mutations and/or reassortment that may have pandemic potential, hence allowing timely implementation of public health interventions and to impose control measures. For example, wastewater or aquatic environment monitoring can be used for tracking and providing early warnings of disease outbreaks^43,44^, and to detect evolutionary adaptations of concern. By combining the latest methods with existing health surveillance frameworks^45^, it is possible to achieve a more comprehensive assessment of AIV outbreaks, thereby protecting nearby wildlife populations by interrupting virus dispersal. Recent studies have detected the HPAI H5N1 virus in wastewater from 10 cities across the United States^46^, indicating that there is a widespread presence of this virus. However, the application of wastewater monitoring remains challenging: the origin of the virus in wastewater is difficult to ascertain, and genome characterization methods for distinguishing AIV from other IAVs endemic in humans are costly. However, sampling from sewage systems near outbreak sites, when combined with epidemiological data is more cost-effective. Recently, Branda et al. have developed a monitoring system by integrated and standardized wastewater data from the WastewaterSCAN platform^47^. The resulting dataset provides crucial information, including the concentration of nucleic acids for H5-related targets in solid samples from municipal sewage. Consequently, wastewater monitoring should be considered as a sentinel surveillance tool. In addition, the utilization of NGS allows comprehensive identification of any pathogens present within the sample, allowing the evaluation of cross-species transmission risks for multiple AIVs simultaneously^6^. For serological surveillance, it is important to monitor the positivity rates across different mammalian hosts in endemic regions, even though it may be limited by factors such as weak host immune response. This is particularly important in light of the current panzoonotic of the H5N1 virus. Of note, to be cost-effective, this comprehensive surveillance should be conducted as risk-based action, focusing on areas where AIV outbreaks occur most frequently, particularly along migratory bird routes during migratory seasons, and for potentially at-risk populations such as farm workers and veterinarians. For resource-poor regions, comprehensive monitoring may not be feasible; instead, regular small-scale active monitoring of birds or conducting targeted surveillance of high-risk populations are more appropriate. In summary, qPCR remains the preferred method for nucleic acid detection; however, its application may be constrained in resource-limited situations. Selecting an appropriate testing method, as summarized in Fig. 2b, should be based on the specific situation and requirements, such as the purpose of the test (pure detection, serotyping, or whole genome sequencing), how soon the results are needed (on-site or laboratory detection), and what the application is (for farmed or wild animals), etc.

In addition to comprehensive monitoring and the implementation of early control measures, the development and application of effective vaccines is an integral part for broader AIV preventive measures within the “One Health” framework. Intense research had been conducted on multiple vaccines platforms—inactivated virus, live-attenuated virus, subunit or recombinant viral protein, nucleic acid (DNA and mRNA), and vector-based. Since H5, H7, and H9 subtypes represent the predominant AIV strains responsible for frequent zoonotic infections, vaccine development for humans should prioritize these subtypes, and is already recommended by the WHO. Of note, pre-pandemic vaccine stockpiles are a critical component of pandemic preparedness, as illustrated by many official contingency plans. For instance, the US maintains a stockpile of vaccines for influenza viruses with pandemic potential, including those targeting specific AIV subtypes, to vaccinate up to 20 million people. In the context of veterinary medicine, it is well-established that vaccination is an effective measure to manage avian influenza outbreaks in poultry and to mitigate and interrupt virus transmission from avian hosts to humans. The current AIV panzoonotic necessitates the implementation of more region-specific vaccination strategies for both humans and animals. International organizations, such as the WHO and the WOAH, should promote and facilitate inter-country cooperation and coordination, to ensure equitable distribution of vaccines for resource-poor regions, thereby fortifying global control efforts of AIV.

## Conclusion and outstanding issues

The recent incursion of H5N1 into new host species (bovine) with accompanying human infections elevates the zoonotic and pandemic risks by AIVs. The need for the One Health framework to mitigate the impact of AIV transmission is becoming more urgent: to advance collaborations among different agencies and to develop advanced diagnostics and vaccines.

Furthermore, there are several outstanding issues that require attention. The foremost is to mitigate the disparity between resource-rich and resource-poor regions regarding diagnostics, surveillance, and preventive measures so that more regions can participate in the monitoring and control of AIVs. Second, even though vaccination is a cost-effective measure against AIV, vaccines exert selective pressure on viral evolution. This situation is exacerbated when different animals are raised together whose vaccination is not universally complete, creating a susceptible population in which mutant viruses can amplify and spread. Third, as migratory birds can travel long distances over remote regions, there is a need for virus surveillance in these inaccessible migration routes. Interestingly, the detection of H5N1 in Antarctica was due to a well-established infrastructure^48^. Therefore, investment to establish a broader network for the surveillance of AIV is needed. Finally, developing new or additional reagents and methods is crucial, allowing the use of serology data for infection status, vaccine efficacy, and overall prevalence, which are important data for a comprehensive risk assessment.
